# Health condition and socioeconomic status mediate the causal effect of reproductive traits on nonalcoholic fatty liver disease: evidence from Mendelian randomization study

**DOI:** 10.3389/fendo.2024.1419964

**Published:** 2024-08-30

**Authors:** Qiaoyun Wang, Liming Wang, Rui Hao, Lijiu Zhang, Wenyan Wang, Lingling Xia

**Affiliations:** ^1^ Department of Infections Disease, the First Affiliated Hospital of Anhui Medical University, Hefei, Anhui, China; ^2^ Department of Gynecology and Obstetrics, the Second Affiliated Hospital of Anhui Medical University, Hefei, Anhui, China; ^3^ Department of Gynecology and Obstetrics, the First Affiliated Hospital of Anhui Medical University, Hefei, Anhui, China; ^4^ Department of Gastroenterology, the Second Affiliated Hospital of Anhui Medical University, Hefei, Anhui, China

**Keywords:** reproductive traits, nonalcoholic fatty liver disease, body mass index, major depression, socioeconomic status, mendelian randomization, mediation

## Abstract

**Background:**

Observational data posits a correlation between reproductive traits and nonalcoholic fatty liver disease (NAFLD), but their causal inference is still unclear. This investigation seeks to elucidate the causal influence of reproductive traits on NAFLD and determine the intervening role of health condition and socioeconomic status in these connections.

**Methods:**

Utilizing a Mendelian Randomization (MR) approach, this research leveraged a comprehensive dataset from the Genome-wide Association Study (GWAS) database. The study incorporated body mass index, major depression, educational level, household income and Townsend deprivation index as intermediary variables. Initially, a bidirectional two-sample MR study was conducted to explore the genetic associations between reproductive traits and NAFLD. Then, two-step MR analyses were implemented to quantify the extent of mediation by these indicators. The weighted inverse variance method was the primary analytical approach, complemented by several sensitivity analyses to affirm the robustness of the MR assumptions. Finally, these findings were validated in the FinnGen research.

**Results:**

The bidirectional MR analysis indicated that earlier reproductive traits (age at menarche, age at first sexual intercourse, and age at first birth) were associated with an elevated risk of NAFLD, absent any evidence of the reverse relationship. Body mass index accounted for 35.64% of the association between premature menarche and NAFLD. Additionally, body mass index, major depression, educational level and household income mediated 41.65%, 14.35%, 37.88%, and 18.59% of the connection between early sexual intercourse and NAFLD, respectively. Similarly, these same variables elucidated 36.36%, 15.58%, 41.56%, and 22.73% of the correlation between younger age at first birth and NAFLD.

**Conclusion:**

Our study elucidated the causal relationships between reproductive traits and NAFLD. Potential underlying mechanisms may involve factors such as body mass index, major depression, educational attainment and household income.

## 1 Introduction

The escalating prevalence of nonalcoholic fatty liver disease (NAFLD), affecting an estimated 30% of the global population (1, 2), cannot be disregarded. Metabolic syndrome, closely associated with obesity, insulin resistance, and hyperlipidemia (3), is recognized as the predominant cause of NAFLD. These conditions contribute to chronic inflammation, poor lipid, and hepatocellular carcinoma (4, 5). Regrettably, there is no approved treatment for NAFLD (6). Hence, it becomes imperative to promptly detect and address the risk elements linked to NAFLD.

Reproductive health and evolutionary adaptability are significantly influenced by female reproductive behaviors. This behavioral aspect encompasses various key factors, such as age at menarche (AAM), age at first sexual intercourse (AFS), age at first birth (AFB), age at last birth (ALB), and age at menopause (AMP). Numerous studies have proposed a correlation between AAM and NAFLD (7–10). Similarly, a meta-analysis evidence indicates that menopause is associated with approximately 2.4 times higher odds of NAFLD (11). Another study has highlighted the impact of menopause on the severity of fibrosis among individuals with non-alcoholic steatohepatitis (12). Nonetheless, the genetic links between AMP and NAFLD remain to be fully understood.

Recent evidence from the NHANES study verified the interaction between AFB and NAFLD (13). Meanwhile, Zuo et al. observed a connection between AFB/ALB and the risk of metabolic syndrome in women (14). However, the causal relationships between these reproductive factors remain uncertain due to constraints in traditional observational research. Moreover, research investigating the correlation between AFS and NAFLD is scarce. Thus, the current understanding of the genetic association between reproductive factors and NAFLD is limited.

Mendelian randomization (MR) analysis has become increasingly popular in genetic research for assessing causal relationships between exposure and outcome variables (15, 16). This method effectively mitigates confounding factors and infers causal relationships by randomly assigning alleles exposed to genetic variation (17, 18). These advantages also extend to intermediary analysis (19, 20). The potential mediating factors can be accurately estimated using the two-step MR approach.

Premature menarche is associated with the occurrence of NAFLD, although there are divergent viewpoints regarding the role of body mass index (BMI) in this association (7–10). Emerging research suggests that major depression (MD) correlates with NAFLD and reproductive behaviors (21–23). In addition, some cross-sectional studies have shown that lower household income and educational levels are associated with an increase the risk of suffering hepatic steatosis in U.S. adolescents (24, 25). However, there is currently no research that fully reveals the relationship between health conditions, socioeconomic status (SES), and reproductive factors. Utilizing a two-step, two-sample Mendelian randomization study, we aimed to investigate the causal effect between reproductive traits and NAFLD and explore the potential roles of health condition and SES.

## 2 Materials and methods

### 2.1 Study design

**Figure 1:**
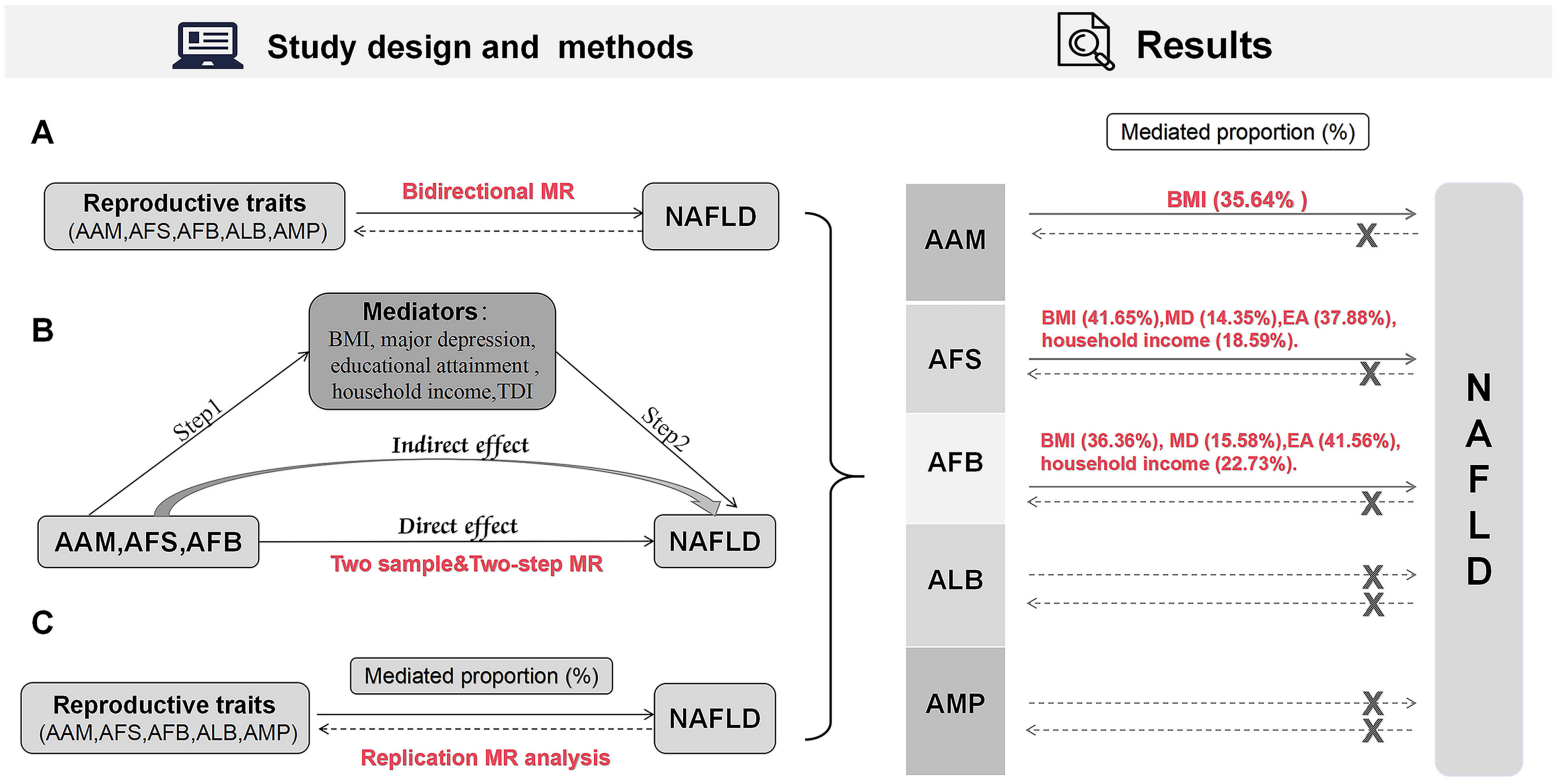
Design and results of the MR analysis. **(A)** Bidirectional MR analysis; **(B)** Mediation MR analysis; **(C)** Replication MR analysis. MR, Mendelian randomization; AAM, age at menarche; AFS, age at first sexual intercourse; AFB, age at first birth; ALB, age at last birth; AMP, age at menopause; NAFLD, nonalcoholic fatty liver disease; BMI, body mass index; MD, major depression; EA, educational attainment; TDI, Townsend deprivation index.

This research aggregated GWAS data and adhered to the MR analysis guidelines as outlined in previous studies (15), while working based on three fundamental assumptions: first, the instrumental variables (IVs) in this study should demonstrate a robust correlation with the exposures. Second, the IVs should be uncontaminated by confounding factors. And finally, the IVs should influence the outcome exclusively through their impact on the exposure. The research design and results are depicted in **Figure 1**.

### 2.2 Data sources and ethics

#### 2.2.1 Reproductive traits

The AAM-related IVs were acquired from a survey encompassing 182,416 female subjects (26). Summary statistics for AFS and AFB were obtained from research conducted by Mills MC (27). This study identified a whopping number of 16,292,532 single nucleotide polymorphisms (SNPs) for AFS and 10,766,720 SNPs for AFB. SNPs for ALB and AMP were downloaded from the IEU Open GWAS database (https://gwas.mrcieu.ac.uk/), with 170,248 and 143,819 individuals, respectively.

#### 2.2.2 NAFLD

Summary-level data for NAFLD were gathered from the largest GWAS meta-analysis, which included 8,434 NAFLD cases and 770,180 controls (discovery stage) (28). The validation dataset consists of 894 cases and 217,898 controls, sourced from the FinnGen Research Project (https://www.finngen.fi/en).

#### 2.2.3 Mediators

The summary data of BMI were obtained from the IEU OpenGWAS database, encompassing 461,460 Europeans. MD related genetic variations were extracted from the Psychiatric Genomes Consortium (PGC), including 500,199 individuals (29).

Three factors of SES were considered at three levels: individual-level SES was assessed by educational attainment (EA), specifically, in years of schooling; household-level SES by average pre-tax household income; and community-level SES by the Townsend deprivation index (TDI) (30). IVs for EA, household income, and TDI were derived from the IEU OpenGWAS project with sample sizes of 99,970, 397,751, and 461,242, respectively.

#### 2.2.4 Ethics

All sample populations are Europeans, which excluded racial variability. Details for datasets are listed in **Table 1**. All data were collected from publicly available databases and published studies, which hence obviated the requirement for any additional ethical clearance.

**Table 1:** Data sources used in the MR analyses for the current study.

Traits	Consortium/Author	Sample size	Population	Year	Pubmed ID
Exposures					
Age at menarche	ReproGen	182,416	European	2014	25231870
Age at first sexual intercourse	Mills MC	182,791	European	2021	34211149
Age at first birth	Mills MC	418,758	European	2021	34211149
Age at last birth	MRC-IEU	170,248	European	2018	/
Age at menopause	MRC-IEU	143,819	European	2018	/
Outcome					
Nonalcoholic fatty liver disease	Ghodsian N	778,614	European	2018	34841290
Nonalcoholic fatty liver disease	FinnGen	218,792	European	2021	/
Mediators					
Body mass index	MRC-IEU	461,460	European	2018	/
Major depression	PGC	500,199	European	2019	30718901
Years of schooling	Within family GWAS consortium	99,970	European	2022	/
Average total household income before tax	MRC-IEU	397,751	European	2018	/
Townsend deprivation index at recruitment	MRC-IEU	462,464	European	2018	/

/ represented PubMed id not retrieved in IEU database.

### 2.3 IVs selection

SNPs with p < 5×10^−8^ were prioritized as the potential IVs. For greater statistical robustness, SNPs (p < 5×10^−7^) were chosen to include more IVs for ALB. And SNPs (p < 5×10^−6^) were used for the reverse analysis (**Supplementary Tables S1**–**S3**). Parameters were set to exclude SNPs affected by linkage disequilibrium (LD), with r^2 =^ 0.001 and a distance of kb=10,000 (31). Subsequently, we excluded weak instruments with F-statistics below 10. The computational formula for F-statistic used here is Beta^2^/SE^2^. Harmonization processes were applied to align datasets on exposure and outcome, minimizing the inclusion of palindromic and ambiguous SNPs with non-concordant alleles (**Supplementary Tables S1**–**S3**).

### 2.4 Mendelian randomization

The standard inverse variance weighted (IVW) method was primarily employed for this estimation. Supplementary analyses included MR-Egger, weighted median, simple mode, and weight mode methods. The IVW is validated a reliable method under the condition that the SNP being used is valid and does not exhibit significant pleiotropy (32). In addition, MR-Egger provides an assessment of potential horizontal pleiotropic effects (33). If no less than 50% of the data from valid instruments is accessible, the weighted median method provides precise and resilient effect estimates (34). The reliability and consistency of the results are verified using both weighted mode and simple mode analyses.

### 2.5 Mediation MR analysis

In a two-step MR process, three β values were obtained: β0, β1, and β2. β0 represents the initial MR of exposures on outcome, β1 represents the impact MR of exposures on mediators, and β2 represents the effect of mediators on outcomes. The calculation of the indirect effect determines the mediation proportion of each mediator, which can be achieved through the formula: (β1×β2)/β0. Delta methods were employed to compute standard errors and confidence intervals (CIs) (35).

### 2.6 Sensitivity analysis

Heterogeneity computation was evaluated by Cochran’s Q statistic (36). The MR results were analyzed using a random-effects model of IVW if the p-value is less than 0.05. If not, a fixed-effects model was used (37). The heterogeneity of IVs was evaluated by examining the p-value of MR-Egger’s intercept. To further validate the potentially abnormal SNPs and address the issue of horizontal pleiotropy, the MR-PRESSO approach was integrated (38).

### 2.7 Replication MR analysis

In order to improve the statistical ability and accuracy of our causal estimation, replication bidirectional MR analysis was performed between reproductive characteristics and NAFLD using the FinnGen Research Project. SNPs with a significance threshold of p < 5×10^−8^ were prioritized as potential IVs. Given the relatively small sample size of NAFLD, the SNP threshold for AAM and MD was adjusted to p < 5×10^−6^. In the reverse analysis, the SNP threshold for NAFLD and AAM analysis was set at p < 5×10^−5^, while the thresholds for other analyses were maintained at p < 5×10^−6^. Finally, the mediation effect was estimated using the replication analysis datasets.

### 2.8 Statistical analysis

All data analyses were performed using the Two Sample MR packages (39) in R version 4.2.1. Results were presented as an odds ratio (OR) with 95%CI per standard deviation. P < 0.05 was deemed to indicate statistical significance.

## 3 Results

### 3.1 Causal effect of reproductive traits on NFLD

#### 3.1.1 Causal effect of AAM and AMP on NAFLD

**Figure 2:**
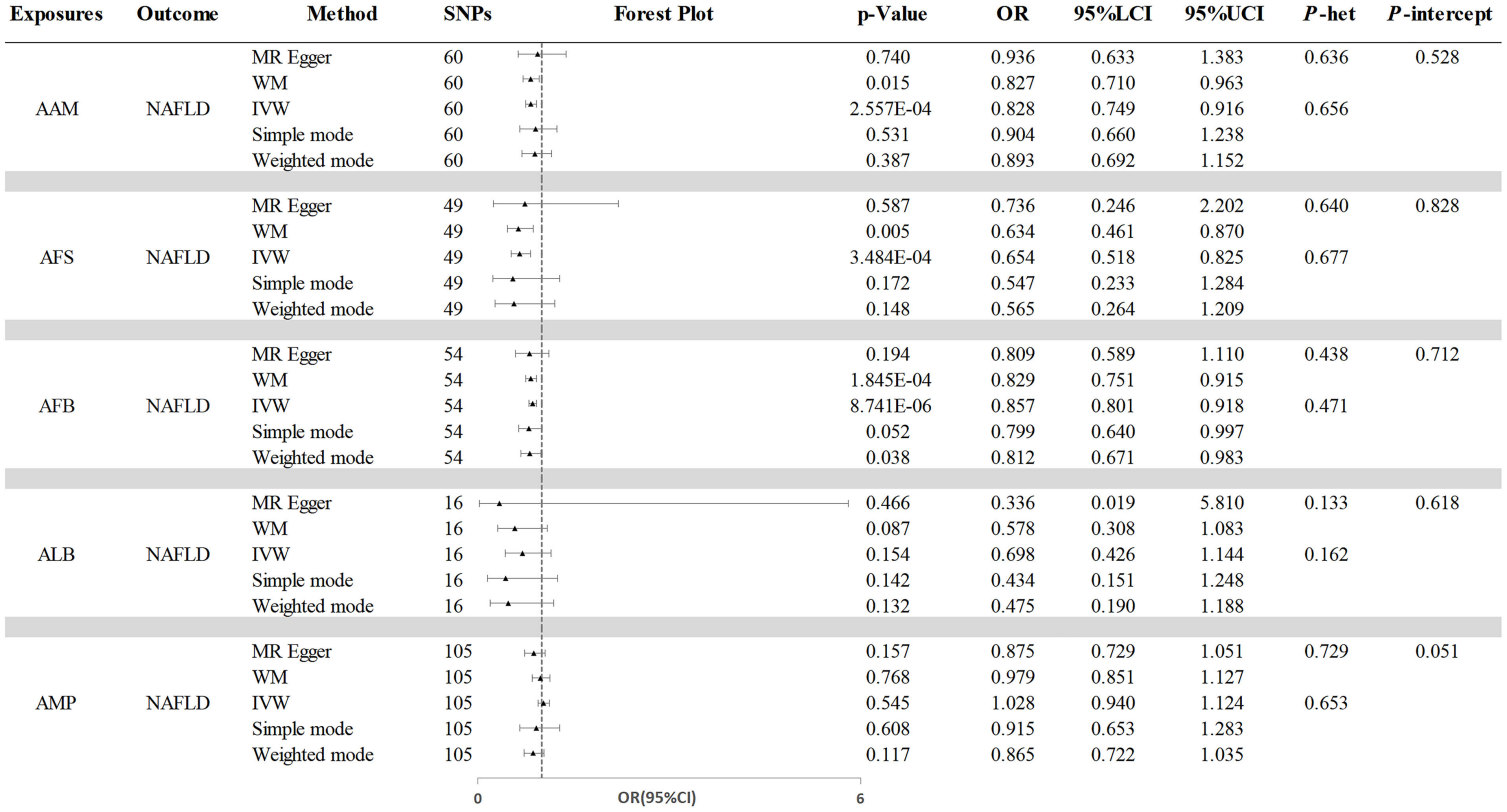
The causal effects of reproductive traits on NAFLD. NAFLD, nonalcoholic fatty liver disease; AAM, age at menarche; AFS, age at first sexual intercourse; AFB, age at first birth; ALB, age at last birth; AMP, age at menopause; WM, weighted median; IVW, inverse-variance weighted; OR, odds ratio; LCI, lower confidence interval; UCI, upper confidence interval; P-het, P value for heterogeneity using Cochran Q test; P-intercept, P value for MR-Egger intercept.

**Figure 3:**
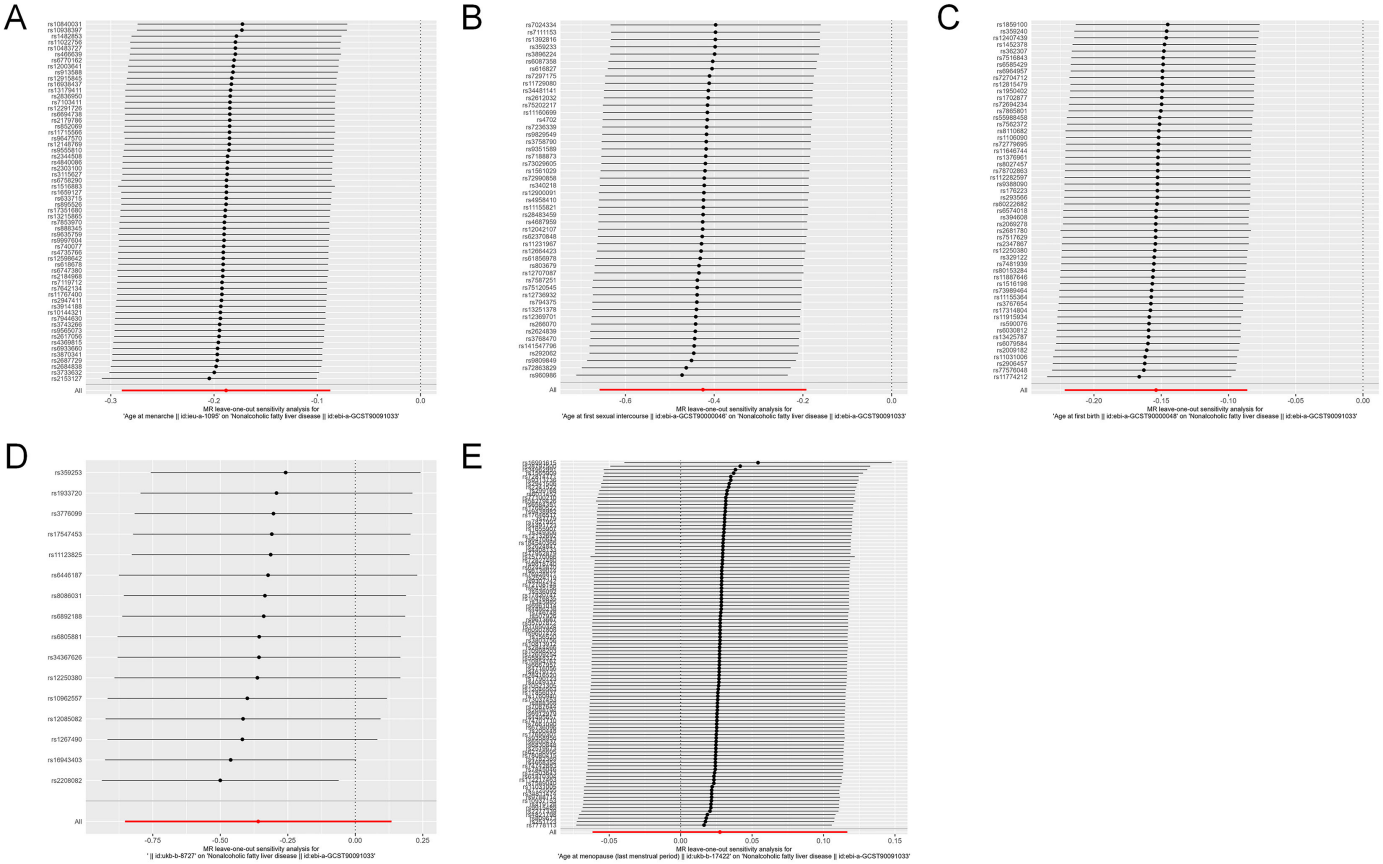
Leave-one-out analysis for causal effects of reproductive traits on NAFLD. **(A)** Leave-one-out analysis plots for age at menarche on NAFLD. **(B)** Leave-one-out analysis plots for age at first sexual intercourse on NAFLD. **(C)** Leave-one-out analysis plots for age at first birth on NAFLD. **(D)** Leave-one-out analysis plots for age at last birth on NAFLD. **(E)** Leave-one-out analysis plots for age at menopause on NAFLD. NAFLD, nonalcoholic fatty liver disease.

The initial analysis explored the impact of AAM on NAFLD. The results, as depicted in **Figure 2**, the IVW analysis indicated a negative causal relationship between AAM and NAFLD with an odds ratio (OR=0.828, 95%CI: 0.749 - 0.916, p=2.557×10^−4^). The Cochran’s Q test revealed the absence of significant heterogeneity (Q=54.110, p =0.656). Moreover, no evidence of pleiotropy was observed (MR-Egger intercept = -5.821×10^−3,^ p =0.528). The robustness of these findings was further validated through the Leave-one-out sensitivity analysis (**Figure 3A**).

Next, we assessed the causal effect of AMP on NAFLD (**Figure 2**). The IVW method indicated no significant effect of AMP on NAFLD (OR=1.028, 95%CI: 0.940 - 1.124, p=0.545). The presence of heterogeneity was not detected by Cochran’s Q test (Q=97.774, p=0.653). And no pleiotropy was observed in the study (MR-Egger intercept=0.008, p=0.051). The Leave-one-out test was shown in **Figure 3E**.

#### 3.1.2 Causal effect of AFS and AFB on NAFLD

Additionally, the IVW analysis revealed a negative causal relationship between AFS and NAFLD (OR=0.654, 95% CI:0.518 - 0.825; p=3.484×10^−4^) (**Figure 2**). Cochran’s Q test did not indicate any heterogeneity (Q=43.018; p=0.677). Additionally, there was no evidence of pleiotropy based on the MR-Egger intercept (MR-Egger intercept = −0.003; p=0.828). A “leave-one-out” test was conducted to confirm the reliability and consistency of the results, which can be seen in **Figure 3B**.

Similarly, the causal link between AFB and NAFLD was assessed using IVW (**Figure 2**), which indicated a negative causal effect (OR=0.857, 95%CI:0.801 - 0.918; p=8.741×10^−6^). Heterogeneity was not observed through Cochran’s Q test (Q =53.078; p=0.471), and no pleiotropy was detected (MR-Egger intercept= 0.004; p=0.712). The results remained stable based on the Leave-one-out test (**Figure 3C**).

#### 3.1.3 Causal effect of ALB on NAFLD

Finally, we investigated the potential association between ALB and NAFLD. As shown in **Figure 2**, IVW results revealing no significant causal connection between ALB and NAFLD (OR=0.698, 95% CI:0.426 - 1.144; p=0.154). Then, neither heterogeneity nor pleiotropy were detected. Leave-one-out analysis for ALB on NAFLD is depicted in **Figure 3D**, with detailed results of sensitivity analyses were depicted in **Supplementary Table S4**.

### 3.2 Causal effects of NAFLD on reproductive traits

**Figure 4:**
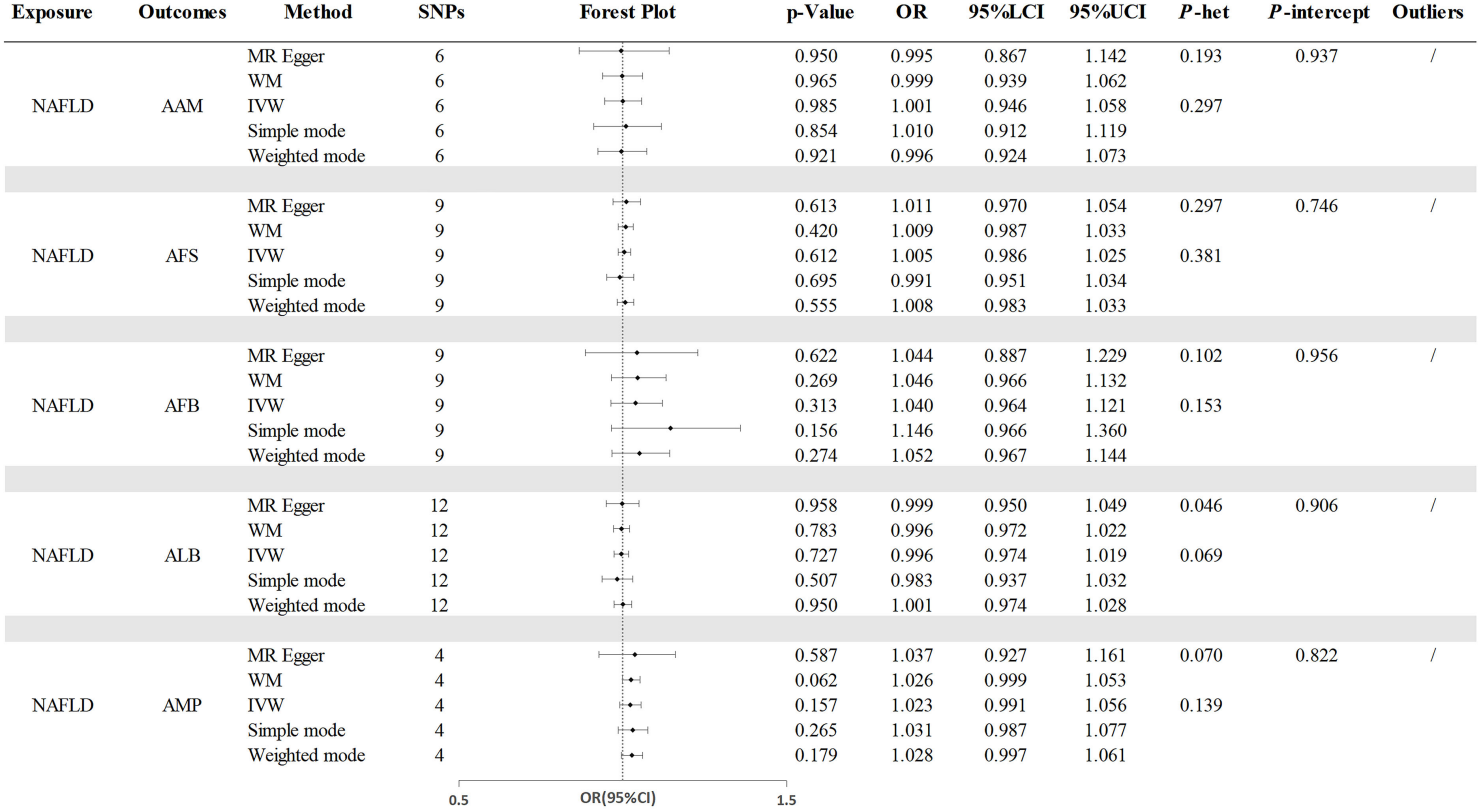
The causal effects of NAFLD on reproductive traits. NAFLD, nonalcoholic fatty liver disease; AAM, age at menarche; AFS, age at first sexual intercourse; AFB, age at first birth; ALB, age at last birth; AMP, age at menopause; WM, weighted median; IVW, inverse-variance weighted; OR, odds ratio; LCI, lower confidence interval; UCI, upper confidence interval; P-het, P value for heterogeneity using Cochran Q test; P-intercept, P value for MR-Egger intercept.

The analysis on MR in reverse revealed that the presence of genetic predisposition towards NAFLD did not influence any of the reproductive characteristics. And no heterogeneity or pleiotropy were detected. The results maintained robust based on the Leave-one-out and MR-PRESSO test (**Figure 4** and **Supplementary Table S5**).

### 3.3 Two-step MR analyses

#### 3.3.1 Causal effects of reproductive traits on mediators

**Figure 5:**
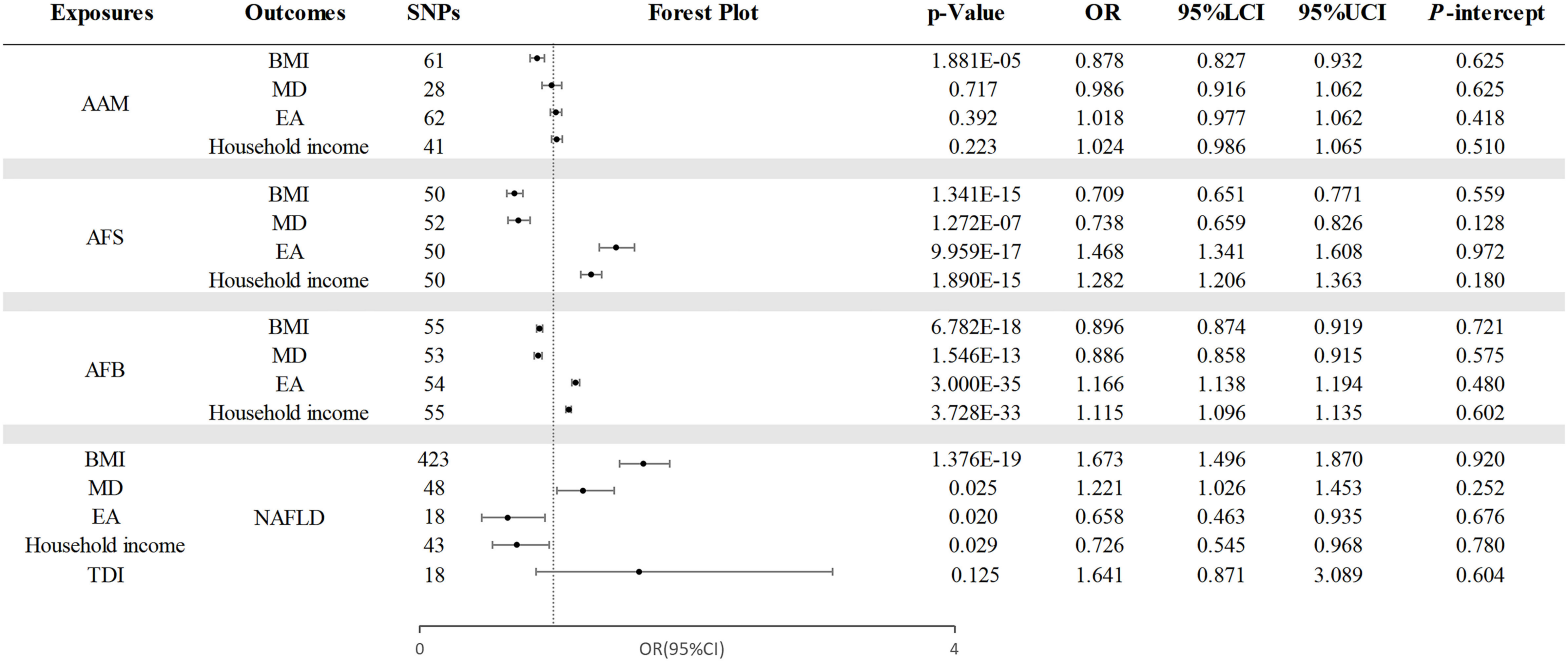
The causal effects of mediating factors in the relationship between AAM, AFS, and AFB and NAFLD. NAFLD, nonalcoholic fatty liver disease; AAM, age at menarche; AFS, age at first sexual intercourse; AFB, age at first birth; BMI, body mass index; MD, major depression; EA, educational attainment; TDI, Townsend deprivation index; OR, odds ratio; LCI, lower confidence interval; UCI, upper confidence interval; P-intercept, P value for MR-Egger intercept.

Our investigation explored the causal relationships between AAM, AFS, and AFB, as ALB and AMP were found to have no causal effect on NAFLD. Additionally, due to the lack of a significant correlation between TDI and NAFLD (OR =1.641, 95% CI =0.871 - 3.089; p=0.125), it was not considered as a mediator in the relationship. **Figure 5** presents the results of the IVW analysis.

Through a two-step MR analysis, we discovered that AAM was causally related to BMI (OR=0.878, 95% CI =0.827 - 0.932; p=1.881×10^−5^). Additionally, AFS exhibited a causal relationship with BMI (OR =0.709, 95% CI =0.651-0.771; p=1.341×10^−15^), MD (OR=0.738, 95% CI=0.659 - 0.826; p =1.272×10^−7^), EA (OR =1.468, 95% CI =1.341 - 1.608; p =9.959×10^−17^), and household income (OR =1.282, 95% CI =1.206 - 1.363; p=1.890×10^−15^). Finally, the causal effects of AFB on mediators is as follows: BMI (OR =0.896, 95% CI: 0.874 - 0.919; p =6.782×10^−18^), MD (OR =0.886, 95% CI =0.858 - 0.915; p =1.546×10^−13^), EA (OR =1.166, 95% CI =1.138 - 1.194; p =3.000×10^−35^), and household income (OR =1.115, 95% CI =1.096 -1.135; p =3.728×10^−33^).

#### 3.3.2 Causal effects of mediators on NAFLD

**Figure 5** also presents the assessment of the potential mediators’ impact on NAFLD. Our findings indicated a positive association between these mediators and NAFLD, encompassing BMI (OR =1.673, 95% CI: 1.496 - 1.870; p =1.376×10^−19^), MD (OR =1.221, 95% CI =1.026 - 1.453; p =0.025), EA (OR =0.658, 95% CI =0.545 - 0.968; p =0.020), and household income (OR =0.726, 95% CI =1.142 - 1.706; p =0.029). Despite the existence of heterogeneity was observed, the random-effects IVW method we selected remains reliable (37). Moreover, the consistency of these associations was confirmed through MR-PRESSO analysis after removing outlier data. the detailed results and sensitivity analyses are listed in **Supplementary Tables S6**–**S9**.

#### 3.3.3 Mediation proportion

As shown in **Table 2**, BMI (35.64%, 95%CI : 18.62%, 54.79%) is considered mediator of the impact of AAM on NAFLD. Four mediating factors revealed the correlation between AFS and NAFLD as follows: BMI (41.65%, 95%CI : 28.94%, 56.24%), MD (14.35%, 95%CI : 1.65%, 29.18%), EA (37.88%, 95%CI : 5.88%, 72.71%), and household income (18.59%, 95%CI : 1.88%, 37.18%). Regarding the link between AFB and NAFLD, the mediators identified include BMI (36.36%, 95%CI : 25.97%, 48.70%), MD (15.58%, 95%CI : 1.95%, 31.17%), EA (41.56%, 95%CI : 6.49%, 77.92%), and household income (22.73%, 95%CI : 2.60%, 44.16%).

**Table 2:** The detailed mediation proportions of mediators.

Exposures	Mediators	Outcome	β0(95% CI)	β1(95% CI)	β2(95%CI)	Mediation effect(95%CI)	Mediated Proportion (%)(95%CI)
AAM	Body mass index	NAFLD	-0.188(-0.289,-0.087)	-0.130(-0.190,-0.070)	0.514(0.403,0.626)	-0.067(-0.103,-0.035)	35.64(18.62,54.79)
AFS	Body mass index	NAFLD	-0.425(-0.658,-0.192)	-0.345(-0.429,-0.260)	0.514(0.403,0.626)	-0.177(-0.239,-0.123)	41.65(28.94,56.24)
	Major depression	NAFLD	-0.425(-0.658,-0.192)	-0.304(-0.417,-0.191)	0.200(0.025,0.374)	-0.061(-0.124,-0.007)	14.35(1.65,29.18)
	Educational attainment	NAFLD	-0.425(-0.658,-0.192)	0.384(0.294,0.475)	-0.419(-0.770,-0.067)	-0.161(-0.309,-0.025)	37.88(5.88,72.71)
	Household income	NAFLD	-0.425(-0.658,-0.192)	0.248(0.187,0.310)	-0.320(-0.607,-0.033)	-0.079(-0.158,-0.008)	18.59(1.88,37.18)
AFB	Body mass index	NAFLD	-0.154(-0.222,-0.086)	-0.109(-0.135,-0.085)	0.514(0.403,0.626)	-0.056(-0.075,-0.040)	36.36(25.97,48.70)
	Major depression	NAFLD	-0.154(-0.222,-0.086)	-0.121(-0.153,-0.089)	0.200(0.025,0.374)	-0.024(-0.048,-0.003)	15.58(1.95,31.17)
	Educational attainment	NAFLD	-0.154(-0.222,-0.086)	0.153(0.129,0.178)	-0.419(-0.770,-0.067)	-0.064(-0.120,-0.010)	41.56(6.49,77.92)
	Household income	NAFLD	-0.154(-0.222,-0.086)	0.109(0.091,0.127)	-0.320(-0.607,-0.033)	-0.035(-0.068,-0.004)	22.73(2.60,44.16)

β0, the initial MR of exposures on outcome; β1, the step one MR of exposures on mediators; β2, the step two MR of mediators on outcome; CI, confidence interval; NAFLD, nonalcoholic fatty liver disease; AAM, age at menarche; AFS, age at first sexual intercourse; AFB, age at first birth. Although the β1 value of AFB and BMI was -0.1096, we marked it as -0.109 here to ensure the accuracy and consistency of the mediation analysis results.

### 3.4 Replication MR analysis

**Figure 6:**
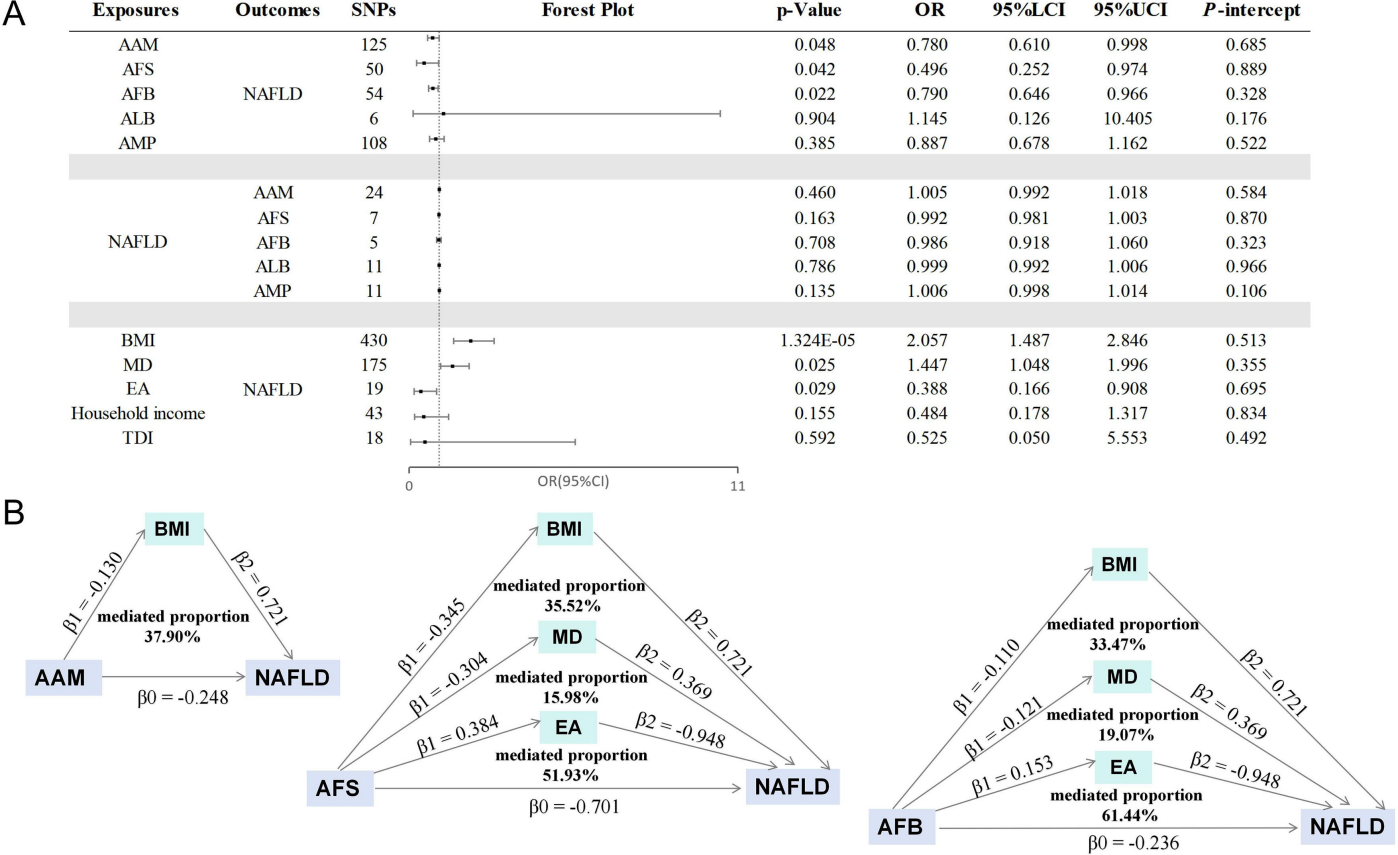
The results of replication MR analysis. **(A)** The causal effects of stepwise MR mediation analysis; **(B)** The mediation proportions of mediators. AAM, age at menarche; AFS, age at first sexual intercourse; AFB, age at first birth; ALB, age at last birth; AMP, age at menopause; NAFLD, nonalcoholic fatty liver disease; BMI, body mass index; MD, major depression; EA, educational attainment; TDI, Townsend deprivation index; LCI, lower confidence interval; UCI, upper confidence interval; P-intercept, P value for MR-Egger intercept.

A replication analysis conducted using the FinnGen database yielded consistent results. The findings revealed that earlier AAM, AFS, and AFB were associated with an increased risk of NAFLD, with no evidence supporting the reverse relationship. **Figure 6** presents the results of the IVW analysis, and no indications of pleiotropy were detected. Specified results and sensitivity analyses are provided in **Supplementary Tables S10**–**S12**.

Subsequently, a two-step mediation analysis was undertaken. Unfortunately, efforts to replicate the link between household income and NAFLD was unsuccessful, attributed to the limited sample size of the Finnish database. Nevertheless, the influence of education level on personal income remains significant, and a clear correlation exists between financial income and NAFLD (24, 25, 40, 41), thereby reinforcing the validity of our findings. As illustrated in **Figure 6**, BMI played a significant role in mediating 37.90% of the relationship between AAM and NAFLD. Moreover, BMI, MD, and EA acted as mediators for 35.52%, 15.98%, and 51.93% of the correlation between AFS and NAFLD. Similarly, the mediators for the association between AFB and NAFLD included BMI (33.47%), MD (19.07%), and EA (61.44%). The results of the stepwise MR analysis can be found in **Supplementary Table S13**.

## 4 Discussion

Our investigation represents the initial endeavor to analyze the causal relationships between reproductive factors (AAM, AFS, AFB, ALB, AMP) and NAFLD using a bidirectional two-sample, two-step MR analysis. Initially, we discovered that individuals experiencing early AAM, AFS, and AFB face an elevated risk of NAFLD development. And there is no evidence establishing a causal connection between ALB, AMP and NAFLD. Furthermore, two-step mediation analyses elucidated that BMI acts as a mediator in the association between AAM and NAFLD. The relationships between AAM, AFS, AFB, and NAFLD are partially mediated by four factors: BMI, MD, EA and household income.

Consistent with prior studies, we found a positive correlation between early menarche age and NAFLD (7–9, 42). Research has demonstrated that early menarche tends to be more prevalent among girls living in urban areas, associated with higher BMI, and intake of high-energy nutrients (43, 44). Moreover, obesity in childhood accounts for much of the cross-national variation in age at menarche (45). Young women who experience early menarche may exhibit pre-existing metabolic abnormalities, such as insulin resistance, poor lipids and blood pressure change (46–49). Therefore, obesity or subsequent weight gain may mediate the linkage between AAM and NAFLD. Our research further indicated that BMI mediates the connection between menarche and NAFLD, which emphasized that we should pay attention to the BMI of adolescents in early menarche age and be alert to the risks of adult obesity and NAFLD.

Then, our research demonstrated an association between AFS and an increased risk of NAFLD. A finding applicable to AFB, as girls who experience AFS at a young age may predispose to earlier AFB. However, the literature currently lacks studies examining the connection between AFS and NAFLD. Previous findings suggest a causal protective effect of education on NAFLD, with individuals of lower socioeconomic status (SES) exhibiting higher incidence rates of NAFLD, particularly in regions such as the United States and Europe (50, 51). It is worth noting that the role of educational attainment and household income on the AFS/AFB-NAFLD relationship also can be interpreted through risky behaviors. Individuals with early reproductive behavior frequently exhibit externalizing behaviors like smoking and alcohol misuse (52–55), which are also recognized predisposing factors for NAFLD (56). In addition, lower educational and income levels among young women who become mothers for the first time often contribute to mental disorders, as well as smoking and alcohol abuse during pregnancy (13, 57). These behaviors further increase the vulnerability to developing NAFLD. Additionally, emerging evidence indicated that early sexual activity is a risk factor for MD, with our MR analysis corroborating this association (58). MD is believed to facilitate NAFLD development through various mechanisms, including inflammation, chronic stress, and gut microbiota (59, 60). Significantly, adolescents experiencing NAFLD exhibited a significant prevalence of clinically diagnosed anxiety and depression (61). Therefore, MD could be a critical metabolic companion in the development of NAFLD caused by AFS. Nevertheless, the exact mechanisms through which AFS impacts BMI remain uncertain, potentially including factors such as AFB.

Previous research has provided evidence linking AFB with an increased risk of NAFLD in women (13). A plausible explanation may involve the influence of weight gain and retention during pregnancy. Even for women who gain weight within expected limits during pregnancy, the accumulated weight during this period and the sustained postpartum weight can contribute to future obesity (62, 63). In obese individuals, abdominal fat accumulation in obese individuals disrupts lipid and glucose metabolism, fostering insulin resistance, thought to be involved in the advancement of NAFLD (64). In terms of the relationship between AFB and MD, a negative correlation has been observed (65). There is a heightened possibility of postpartum depression (PPD) among younger mothers due to a notable decline observed in all opregnanolone levels (66, 67). Our MR analysis further supports the causal influence of early AFB on increasing PPD risk, thereby enhancing the likelihood of NAFLD. Therefore, it is crucial to focus on women’s reproductive health to reduce the incidence of NAFLD.

Numerous factors, including physiological, genetic, environmental, and social influences, contribute to the development of NAFLD (68). Research indicates that individuals from lower socioeconomic backgrounds are disproportionately affected by liver disease, underscoring it as a significant issue of health inequality (69). A cohort study conducted in Iran has demonstrated that a more vulnerable SES is associated with an increased risk of NAFLD (70). Furthermore, a protective effect of higher educational attainment on the risk of NAFLD has been reported (71). Our findings also suggest that social status plays a critical role in the relationship between AFS/AFB and NAFLD. These insights are crucial and imply that guidelines for the prevention and management of NAFLD should be formulated with consideration for the disparities among different socioeconomic status groups.

Our study is characterized by several strengths. Firstly, we introduced a total of five distinct reproductive traits for the first time. The examination of these traits and their correlation with NAFLD was rigorously analyzed using extensive data from many GWAS. By integrating genetic instruments and deploying diverse MR approaches, our investigation enables a comprehensive investigation into the causal inference between reproductive characteristics and the susceptibility to NAFLD. Furthermore, our application of a two-step MR analysis, thereby enhancing our understanding of the underlying mechanisms and provided solid evidence to substantiate prevention strategies. This pioneering effort holds substantial significance in understanding the potential impact of female reproductive characteristics on the risk of NAFLD.

This research acknowledges specific restrictions that must be considered. Firstly, our study was confined to participants of European descent. Further studies should include a broader ethnic range to validate the universality of our findings. Secondly, the reliance on recall data for reproductive age introduces the possibility of bias. This dependence on memory has the potential to introduce recollection partiality, which must be considered while interpreting the outcomes (72, 73). Thirdly, the utilization of GWAS data restricted our ability to investigate potential non-linear connections or variations in stratification effects relating to age or gender. Meanwhile, the sample size from the Finnish database constrained the outcomes of our replication analysis, highlighting the need for larger datasets in future studies. Although we observed pleiotropy in the causal effect of AAM on NAFLD risk in our repeated MR analysis, the magnitude and direction of this causal effect remained consistent with the findings from the primary analysis. In conjunction with previous literature reports, we believe that these results are still significant (74). Finally, given that NAFLD encompasses multiple subtypes, and whether the subtype analysis results are consistent with our research requires more study to understand how reproductive characteristics modulate the risk of NAFLD and its subtypes.

In conclusion, this groundbreaking research indicates early AAM, AFS, and AFB as risk factors for NAFLD. Factors such as BMI, MD, educational level, and house income may mediate these causal relationships, offering valuable insights for targeting interventions at obesity, mental health, and educational disparities to mitigate NAFLD’s burden.

## Data Availability

The original contributions presented in the study are included in the article/**Supplementary Material**. Further inquiries can be directed to the corresponding author/s.

## References

[1] Younossi ZM Golabi P Paik JM Henry A Van Dongen C Henry L. The global epidemiology of nonalcoholic fatty liver disease (NAFLD) and nonalcoholic steatohepatitis (NASH): a systematic review. Hepatology. (2023) 77:1335–47. doi: 10.1097/HEP.0000000000000004 PMC10026948 36626630

[2] Estes C Anstee QM Arias-Loste MT Bantel H Bellentani S Caballeria J. Modeling NAFLD disease burden in China, France, Germany, Italy, Japan, Spain, United Kingdom, and United States for the period 2016-2030. J Hepatol. (2018) 69:896–904. doi: 10.1016/j.jhep.2018.05.036 29886156

[3] Younossi ZM. Non-alcoholic fatty liver disease - A global public health perspective. J Hepatol. (2019) 70:531–44. doi: 10.1016/j.jhep.2018.10.033 30414863

[4] Lange NF Radu P Dufour JF. Prevention of NAFLD-associated HCC: Role of lifestyle and chemoprevention. J Hepatol. (2021) 75:1217–27. doi: 10.1016/j.jhep.2021.07.025 34339764

[5] Huang DQ El-Serag HB Loomba R. Global epidemiology of NAFLD-related HCC: trends, predictions, risk factors and prevention. Nature reviews. Gastroenterol Hepatol. (2021) 18:223–38. doi: 10.1038/s41575-020-00381-6 PMC8016738 33349658

[6] Powell EE Wong VW Rinella M. Non-alcoholic fatty liver disease. Lancet (London England). (2021) 397:2212–24. doi: 10.1016/S0140-6736(20)32511-3 33894145

[7] Ryu S Chang Y Choi Y Kwon MJ Kim CW Yun KE. Age at menarche and non-alcoholic fatty liver disease. J Hepatol. (2015) 62:1164–70. doi: 10.1016/j.jhep.2014.11.041 25500721

[8] Mueller NT Pereira MA Demerath EW Dreyfus JG MacLehose RF Carr JJ. Earlier menarche is associated with fatty liver and abdominal ectopic fat in midlife, independent of young adult BMI: The CARDIA study. Obes (Silver Spring). (2015) 23:468–74. doi: 10.1002/oby.v23.2 PMC4310794 25521620

[9] Li K Yin J Qin Z Ma B He R Zhuoma D. Age at menarche and metabolic dysfunction-associated fatty liver disease: Evidence from a large population-based epidemiological study in Southwest China. Prev Med. (2023) 177:107776. doi: 10.1016/j.ypmed.2023.107776 37951543

[10] Lu J Zhang J Du R Wang T Xu M Xu Y. Age at menarche is associated with the prevalence of non-alcoholic fatty liver disease later in life. J Diabetes. (2017) 9:53–60. doi: 10.1111/1753-0407.12379 26800495

[11] Jaroenlapnopparat A Charoenngam N Ponvilawan B Mariano M Thongpiya J Yingchoncharoen P. Menopause is associated with increased prevalence of nonalcoholic fatty liver disease: a systematic review and meta-analysis. Menopause. (2023) 30:348–54. doi: 10.1097/GME.0000000000002133 36728528

[12] Robeva R Mladenović D Vesković M Hrnčić D Bjekić-Macut J Stanojlović O. The interplay between metabolic dysregulations and non-alcoholic fatty liver disease in women after menopause. Maturitas. (2021) 151:22–30. doi: 10.1016/j.maturitas.2021.06.012 34446275

[13] Yang HH Chen GC Zhou MG Xie LF Jin YY Chen HT. Association of age at first birth and risk of non-alcoholic fatty liver disease in women: evidence from the NHANES. Hepatol Int. (2023) 17:303–12. doi: 10.1007/s12072-022-10429-1 36227515

[14] Zuo R Ge Y Xu J He L Liu T Wang B. The association of female reproductive factors with risk of metabolic syndrome in women from NHANES 1999-2018. BMC Public Health. (2023) 23:2306. doi: 10.1186/s12889-023-17207-0 37990201 PMC10664376

[15] Skrivankova VW Richmond RC Woolf BAR Yarmolinsky J Davies NM Swanson SA. Strengthening the reporting of observational studies in epidemiology using mendelian randomization: the STROBE-MR statement. JAMA. (2021) 326:1614–21. doi: 10.1001/jama.2021.18236 34698778

[16] Tin A Köttgen A. Mendelian randomization analysis as a tool to gain insights into causes of diseases: A primer. J Am Soc Nephrol: JASN. (2021) 32:2400–7. doi: 10.1681/ASN.2020121760 PMC8722812 34135084

[17] Weng H Li H Zhang Z Zhang Y Xi L Zhang D. Association between uric acid and risk of venous thromboembolism in East Asian populations: a cohort and Mendelian randomization study. Lancet Regional Health Western Pacific. (2023) 39:100848. doi: 10.1016/j.lanwpc.2023.100848 37565068 PMC10410163

[18] Li L Sun Y Luo J Liu M. Circulating immune cells and risk of osteosarcoma: a Mendelian randomization analysis. Front Immunol. (2024) 15:1381212. doi: 10.3389/fimmu.2024.1381212 39081321 PMC11286390

[19] Hu X Zhao J Lin Z Wang Y Peng H Zhao H. Mendelian randomization for causal inference accounting for pleiotropy and sample structure using genome-wide summary statistics. Proc Natl Acad Sci United States America. (2022) 119:e2106858119. doi: 10.1073/pnas.2106858119 PMC9282238 35787050

[20] Carter AR Sanderson E Hammerton G Richmond RC Davey Smith G Heron J. Mendelian randomisation for mediation analysis: current methods and challenges for implementation. Eur J Epidemiol. (2021) 36:465–78. doi: 10.1007/s10654-021-00757-1 PMC8159796 33961203

[21] Cai H Zhang R Zhao C Wang Y Tu X Duan W. Associations of depression score with metabolic dysfunction-associated fatty liver disease and liver fibrosis. J Affect Disord. (2023) 334:332–6. doi: 10.1016/j.jad.2023.04.093 37142003

[22] Lee JW Park SH. Association between depression and nonalcoholic fatty liver disease: Contributions of insulin resistance and inflammation. J Affect Disord. (2021) 278:259–63. doi: 10.1016/j.jad.2020.09.073 32977263

[23] Yu Y Hou L Wu Y Yu Y Liu X Wu S. Causal associations between female reproductive behaviors and psychiatric disorders: a lifecourse Mendelian randomization study. BMC Psychiatry. (2023) 23:799. doi: 10.1186/s12888-023-05203-y 37915018 PMC10621101

[24] Paik JM Duong S Zelber-Sagi S Lazarus JV Henry L Younossi ZM. Food insecurity, low household income, and low education level increase the risk of having metabolic dysfunction associated fatty liver disease (MASLD) among adolescents in the United States. Am J Gastroenterol. (2024) 119(6):1089–1101. doi: 10.14309/ajg.0000000000002749 38477467

[25] Tang M Liu M Zhang Y Xie R. Association of family income to poverty ratio and vibration-controlled transient elastography quantified degree of hepatic steatosis in U.S. adolescents. Front Endocrinol (Lausanne). (2023) 14:1160625. doi: 10.3389/fendo.2023.1160625 37033220 PMC10079211

[26] Perry JR Day F Elks CE Sulem P Thompson DJ Ferreira T. Parent-of-origin-specific allelic associations among 106 genomic loci for age at menarche. Nature. (2014) 514:92–7. doi: 10.1038/nature13545 PMC4185210 25231870

[27] Mills MC Tropf FC Brazel DM van Zuydam N Vaez A eQTLGen Consortium. Identification of 371 genetic variants for age at first sex and birth linked to externalising behaviour. Nat Hum Behav. (2021) 5:1717–30. doi: 10.1038/s41562-021-01135-3 PMC7612120 34211149

[28] Anstee QM Darlay R Cockell S Meroni M Govaere O Tiniakos D. Genome-wide association study of non-alcoholic fatty liver and steatohepatitis in a histologically characterised cohort(☆). J Hepatol. (2020) 73:505–15. doi: 10.1016/j.jhep.2020.04.003 32298765

[29] Howard DM Adams MJ Clarke TK Hafferty JD Gibson J Shirali M. Genome-wide meta-analysis of depression identifies 102 independent variants and highlights the importance of the prefrontal brain regions. Nat Neurosci. (2019) 22:343–52. doi: 10.1038/s41593-018-0326-7 PMC6522363 30718901

[30] Peng L Wen WP. Socioeconomic status and asthma: A bidirectional Mendelian randomization study. World Allergy Organ J. (2023) 16:100790. doi: 10.1016/j.waojou.2023.100790 37484875 PMC10362521

[31] Myers TA Chanock SJ Machiela MJ. LDlinkR: an R package for rapidly calculating linkage disequilibrium statistics in diverse populations. Front Genet. (2020) 11:157. doi: 10.3389/fgene.2020.00157 32180801 PMC7059597

[32] Burgess S Butterworth A Thompson SG. Mendelian randomization analysis with multiple genetic variants using summarized data. Genet Epidemiol. (2013) 37:658–65. doi: 10.1002/gepi.21758 PMC4377079 24114802

[33] Bowden J Davey Smith G Burgess S. Mendelian randomization with invalid instruments: effect estimation and bias detection through Egger regression. Int J Epidemiol. (2015) 44:512–25. doi: 10.1093/ije/dyv080 PMC4469799 26050253

[34] Bowden J Davey Smith G Haycock PC Burgess S. Consistent estimation in mendelian randomization with some invalid instruments using a weighted median estimator. Genet Epidemiol. (2016) 40:304–14. doi: 10.1002/gepi.21965 PMC4849733 27061298

[35] Thompson JR Minelli C Del Greco MF. Mendelian randomization using public data from genetic consortia. Int J Biostatistics. (2016) 12. doi: 10.1515/ijb-2015-0074 27092657

[36] Cohen JF Chalumeau M Cohen R Korevaar DA Khoshnood B Bossuyt PM. Cochran’s Q test was useful to assess heterogeneity in likelihood ratios in studies of diagnostic accuracy. J Clin Epidemiol. (2015) 68:299–306. doi: 10.1016/j.jclinepi.2014.09.005 25441698

[37] Bowden J Del Greco MF Minelli C Davey Smith G Sheehan N Thompson J. A framework for the investigation of pleiotropy in two-sample summary data Mendelian randomization. Stat Med. (2017) 36:1783–802. doi: 10.1002/sim.7221 PMC5434863 28114746

[38] Verbanck M Chen CY Neale B Do R. Detection of widespread horizontal pleiotropy in causal relationships inferred from Mendelian randomization between complex traits and diseases. Nat Genet. (2018) 50:693–8. doi: 10.1038/s41588-018-0099-7 PMC6083837 29686387

[39] Hemani G Zheng J Elsworth B Wade KH Haberland V Baird D. The MR-Base platform supports systematic causal inference across the human phenome. Elife. (2018) 7:e34408. doi: 10.7554/eLife.34408 29846171 PMC5976434

[40] Ren Z Bosma H Wesselius A Eussen SJPM Kooi ME van der Kallen CJH. Traditional lifestyle factors partly mediate the association of socioeconomic position with intrahepatic lipid content: The Maastricht study. JHEP Reports: Innovation Hepatol. (2023) 5:100855. doi: 10.1016/j.jhepr.2023.100855 PMC10522893 37771365

[41] Liu L Lin J Yin M Liu L Gao J Liu X. Association of the fat mass index with hepatic steatosis and fibrosis: evidence from NHANES 2017-2018. Sci Rep. (2024) 14(1):6943. doi: 10.1038/s41598-024-57388-1 38521854 PMC10960854

[42] Wang J Wu AH Stanczyk FZ Porcel J Noureddin M Terrault NA. Associations between reproductive and hormone-related factors and risk of nonalcoholic fatty liver disease in a multiethnic population. Clin Gastroenterol Hepatol: Off Clin Pract J Am Gastroenterol Assoc. (2021) 19:1258–1266.e1251. doi: 10.1016/j.cgh.2020.08.012 PMC7878579 32801014

[43] Gill D Brewer CF Del Greco MF Sivakumaran P Bowden J Sheehan NA. Age at menarche and adult body mass index: a Mendelian randomization study. Int J Obes (2005). (2018) 42:1574–81. doi: 10.1038/s41366-018-0048-7 29549348

[44] Meng X Li S Duan W Sun Y Jia C. Secular trend of age at menarche in chinese adolescents born from 1973 to 2004. Pediatrics. (2017) 140(2):e20170085. doi: 10.1542/peds.2017-0085 28716824 PMC5527668

[45] Currie C Ahluwalia N Godeau E Nic Gabhainn S Due P Currie DB. Is obesity at individual and national level associated with lower age at menarche? Evidence from 34 countries in the Health Behaviour in School-aged Children Study. J Adolesc Health: Off Publ Soc Adolesc Med. (2012) 50:621–6. doi: 10.1016/j.jadohealth.2011.10.254 22626490

[46] Bubach S Horta BL Gonçalves H Assunção MCF. Early age at menarche and metabolic cardiovascular risk factors: mediation by body composition in adulthood. Sci Rep. (2021) 11:148. doi: 10.1038/s41598-020-80496-7 33420216 PMC7794383

[47] Zhang Z Hu X Yang C Chen X. Early age at menarche is associated with insulin resistance: a systemic review and meta-analysis. Postgraduate Med. (2019) 131:144–50. doi: 10.1080/00325481.2019.1559429 30560708

[48] Remsberg KE Demerath EW Schubert CM Chumlea WC Sun SS Siervogel RM. Early menarche and the development of cardiovascular disease risk factors in adolescent girls: the Fels Longitudinal Study. J Clin Endocrinol Metab. (2005) 90:2718–24. doi: 10.1210/jc.2004-1991 15728207

[49] Wang G Radovick S Buckley JP Hauser R Williams PL Hong X. Plasma insulin concentration in newborns and children and age at menarche. Diabetes Care. (2023) 46:1231–8. doi: 10.2337/dc22-2017 PMC10234748 37018448

[50] Aerts M Rosseel Z De Waele E. The evolution in non-alcoholic fatty liver disease patients’ Profile and the associated sustainable challenges: A multidisciplinary perspective. Nutrients. (2024) 16:1584. doi: 10.3390/nu16111584 38892517 PMC11174485

[51] Koutny F Aigner E Datz C Gensluckner S Maieron A Mega A. Relationships between education and non-alcoholic fatty liver disease. Eur J Internal Med. (2023) 118:98–107. doi: 10.1016/j.ejim.2023.07.039 37541922

[52] David AT Sharma V Bittencourt L Gurka KK Perez-Carreño JG Lopez-Quintero C. Exploring the associations between serious psychological distress and the quantity or frequency of tobacco, alcohol, and cannabis use among pregnant women in the United States. Prev Med. (2023) 177:107770. doi: 10.1016/j.ypmed.2023.107770 37951544 PMC11099898

[53] Yeo JH Park H Kim EY. Predictors of the timing of sexual intercourse initiation among adolescents in South Korea. J Community Health. (2019) 44:580–6. doi: 10.1007/s10900-018-00605-6 30604219

[54] Shayo FK Kalomo MH. Prevalence and correlates of sexual intercourse among sexually active in-school adolescents: an analysis of five sub-Sahara African countries for the adolescent’s sexual health policy implications. BMC Public Health. (2019) 19:1285. doi: 10.1186/s12889-019-7632-1 31606038 PMC6790023

[55] Zhang Y Wang C Liang M. A latent class analysis of sexual behavior and associations with sex education, smoking, drinking, and pornography use among chinese youth. Arch Sexual Behav. (2022) 51:1351–61. doi: 10.1007/s10508-021-02091-9 34750778

[56] Yuan S Chen J Ruan X Sun Y Zhang K Wang X. Smoking, alcohol consumption, and 24 gastrointestinal diseases: Mendelian randomization analysis. Elife. (2023) 12:e84051. doi: 10.7554/eLife.84051 36727839 PMC10017103

[57] Younossi ZM Zelber-Sagi S Henry L Gerber LH. Lifestyle interventions in nonalcoholic fatty liver disease. Nat Rev Gastroenterol Hepatol. (2023) 20:708–22. doi: 10.1038/s41575-023-00800-4 37402873

[58] Lu Z Sun Y Liao Y Kang Z Feng X Zhao G. Identifying causal associations between early sexual intercourse or number of sexual partners and major depressive disorders: A bidirectional two-sample Mendelian randomization analysis. J Affect Disord. (2023) 333:121–9. doi: 10.1016/j.jad.2023.04.079 37086791

[59] Shao Q Wu Y Ji J Xu T Yu Q Ma C. Interaction mechanisms between major depressive disorder and non-alcoholic fatty liver disease. Front Psychiatry. (2021) 12:711835. doi: 10.3389/fpsyt.2021.711835 34966296 PMC8710489

[60] Shea S Lionis C Kite C Atkinson L Chaggar SS Randeva HS. Non-alcoholic fatty liver disease (NAFLD) and potential links to depression, anxiety, and chronic stress. Biomedicines. (2021) 9(11):1697. doi: 10.3390/biomedicines9111697 34829926 PMC8615558

[61] Noon SL D’Annibale DA Schwimmer MH Shiels J Arin J Durelle J. Incidence of depression and anxiety in a cohort of adolescents with nonalcoholic fatty liver disease. J Pediatr Gastroenterol Nutr. (2021) 72:579–83. doi: 10.1097/MPG.0000000000003024 PMC8815421 33346572

[62] Gore SA Brown DM West DS. The role of postpartum weight retention in obesity among women: a review of the evidence. Ann Behav Med: Publ Soc Behav Med. (2003) 26:149–59. doi: 10.1207/S15324796ABM2602_07 14534032

[63] Endres LK Straub H McKinney C Plunkett B Minkovitz CS Schetter CD. Postpartum weight retention risk factors and relationship to obesity at 1 year. Obstetrics Gynecol. (2015) 125:144–52. doi: 10.1097/AOG.0000000000000565 PMC4286308 25560116

[64] Tilg H Moschen AR. Insulin resistance, inflammation, and non-alcoholic fatty liver disease. Trends Endocrinol Metabol: TEM. (2008) 19:371–9. doi: 10.1016/j.tem.2008.08.005 18929493

[65] Mirowsky J Ross CE. Depression, parenthood, and age at first birth. Soc Sci Med. (2002) 54:1281–98. doi: 10.1016/S0277-9536(01)00096-X 11989963

[66] Ou Z Gao Z Wang Q Lin Y Ye D. Association between age at first birth and postpartum depression: A two-sample mendelian randomization analysis. Heliyon. (2023) 9:e20500. doi: 10.1016/j.heliyon.2023.e20500 37790979 PMC10543215

[67] Wang Z Lu J Weng W Fu J Zhang J. Women’s reproductive traits and major depressive disorder: A two-sample Mendelian randomization study. J Affect Disord. (2023) 326:139–46. doi: 10.1016/j.jad.2023.01.063 36682697

[68] Talens M Tumas N Lazarus JV Benach J Pericàs JM. What do we know about inequalities in NAFLD distribution and outcomes? A scoping review. J Clin Med. (2021) 10:5019. doi: 10.3390/jcm10215019 34768539 PMC8584385

[69] Williams CD Stengel J Asike MI Torres DM Shaw J Contreras M. Prevalence of nonalcoholic fatty liver disease and nonalcoholic steatohepatitis among a largely middle-aged population utilizing ultrasound and liver biopsy: a prospective study. Gastroenterology. (2011) 140:124–31. doi: 10.1053/j.gastro.2010.09.038 20858492

[70] Sadeghianpour Z Cheraghian B Farshchi HR Asadi-Lari M. Non-alcoholic fatty liver disease and socioeconomic determinants in an Iranian cohort study. BMC Gastroenterol. (2023) 23:350. doi: 10.1186/s12876-023-02964-4 37814220 PMC10561474

[71] Ren Z Wesselius A Stehouwer CDA Brouwers MCGJ. Relationship between educational attainment and non-alcoholic fatty liver disease: A two-sample Mendelian randomization study. Digestive Liver Dis: Off J Ital Soc Gastroenterol Ital Assoc Study Liver. (2024) 56:565–70. doi: 10.1016/j.dld.2023.11.040 38104027

[72] Cooper R Blell M Hardy R Black S Pollard TM Wadsworth ME. Validity of age at menarche self-reported in adulthood. J Epidemiol Community Health. (2006) 60:993–7. doi: 10.1136/jech.2005.043182 PMC2465480 17053289

[73] Graham CA Catania JA Brand R Duong T Canchola JA. Recalling sexual behavior: a methodological analysis of memory recall bias via interview using the diary as the gold standard. J Sex Res. (2003) 40:325–32. doi: 10.1080/00224490209552198 14735406

[74] Liu D Gao X Pan XF Zhou T Zhu C Li F. The hepato-ovarian axis: genetic evidence for a causal association between non-alcoholic fatty liver disease and polycystic ovary syndrome. BMC Med. (2023) 21:62. doi: 10.1186/s12916-023-02775-0 36800955 PMC9940436

